# Controversies regarding encapsulated papillary carcinoma of the breast: an approach to evaluation and categorisation

**DOI:** 10.1111/his.15310

**Published:** 2024-08-29

**Authors:** Emad A Rakha, Cecily Quinn

**Affiliations:** ^1^ Pathology Department School of Medicine, The University of Nottingham, Nottingham University Hospitals NHS Trust Nottingham UK; ^2^ Department of Pathology Hamad Medical Corporation Doha Qatar; ^3^ Irish National Breast Screening Programme and Department of Histopathology St. Vincent's University Hospital Dublin Ireland; ^4^ School of Medicine, University College Dublin Ireland

**Keywords:** breast cancer, challenges, diagnosis, encapsulated papillary carcinoma

## Abstract

Malignant papillary lesions, and in particular, encapsulated papillary carcinoma (EPC) of the breast, continue to present diagnostic challenges for the practising pathologist. In addition to the relative rarity of these lesions, the lack of evidence‐based diagnostic criteria, differences in the biological characteristics, and the clinical behaviour of *in situ* and invasive forms, variable use of immunohistochemical markers, and overlap with other tumour types including high‐grade circumscribed forms of invasive breast carcinomas has resulted in diagnostic discordance with potentially significant clinical and management implications. Pathologists should be familiar with the range of morphology observed in malignant papillary tumours, EPC, and EPC‐like tumours and the existence of tumours with overlapping features. In this review we summarize the common diagnostic pitfalls in malignant papillary tumours and provide an approach to the diagnostic evaluation and categorisation of these enigmatic entities.

## Graphical abstract

This review summarizes the common diagnostic pitfalls in malignant papillary tumours of the breast, with particular focus on encapsulated papillary carcinoma, and provide an approach to the diagnostic evaluation and categorisation of these enigmatic entities.

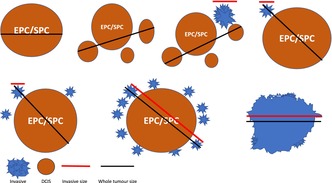

**Abbreviations**
CK: cytokeratin
DCIS: Ductal carcinoma in situ
EPC: Encapsulated papillary carcinoma
ER: Oestrogen receptor
IBC: Invasive breast carcinoma
IHC: Immunohistochemistry
LCIS: Lobular Carcinoma in situ
MECs: Myoepithelial cells
NST: No special type
SLN: Sentinel lymph node
SPC: Solid papillary carcinom
WHO: World Health Organisation

## Introduction

Papillary lesions of the breast comprise a heterogeneous group of breast tumours characterized by epithelial cell proliferation supported by fibrovascular connective tissue cores.^1^, ^2^, ^3^, ^4^, ^5^ Papillary morphogenesis is not a feature of normal breast tissue and its development is poorly understood. Papillary lesions are broadly classified according to the nature and morphology of the proliferating epithelial cells, the presence or absence of myoepithelial cells (MECs) lining the fibrovascular cores and at the peripheral epithelial stroma interface, the outline of the lesion, and its location within the glandular duct system.^6^, ^7^, ^8^, ^9^, ^10^ Papillary lesions, including benign, benign involved by a neoplastic epithelial cell proliferation and malignant entities, often pose diagnostic challenges, with interobserver variation in categorisation^11^ and comprise one of the most frequent type of breast lesions referred for external specialist opinion.^12^ Diagnostic challenges presented by papillary lesions in general have been addressed in previous reviews^7^, ^8^, ^9^, ^10^ and a comprehensive update provided in the World Health Organization (WHO) Classification of Tumours (Blue Book), 5th Edition Breast Tumours.^13^ However, some challenges and pitfalls continue to cause difficulty in daily practice. These include the classification of encapsulated papillary carcinoma (EPC) into *in situ* and invasive disease, interpretation of immunohistochemical (IHC) markers, and the distinction of EPC from EPC‐like circumscribed high‐grade invasive breast carcinoma (IBC).^11^

In this review we focus on diagnostic challenges that are encountered in the precise classification of malignant papillary tumours with particular focus on EPC. These challenges frequently stem from overlapping morphological and IHC features in various entities and the lack of reliable criteria for accurate and reproducible classification. We outline our approach to the evaluation of these tumours, with the aim of improving consistency in reporting and promoting a pragmatic approach to the classification of borderline lesions to avoid over‐ or undertreatment of patients.

### Encapsulated papillary carcinoma

**Figure 1:**
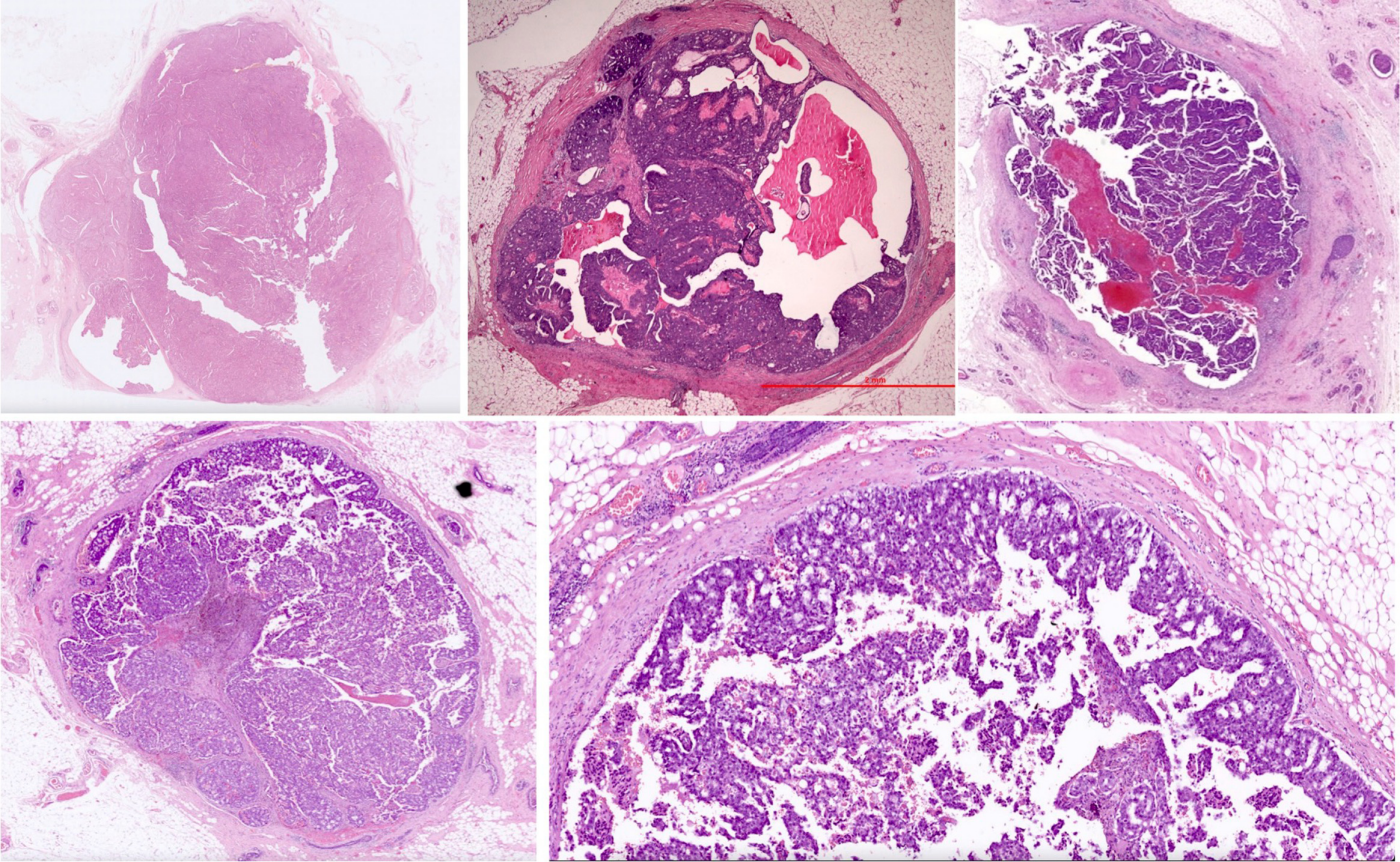
Examples of encapsulated papillary carcinoma (EPC) demonstrating a cystic papillary architecture, circumscribed margin, and peripheral thick fibrous capsules. [Color figure can be viewed at wileyonlinelibrary.com]

**Figure 2:**
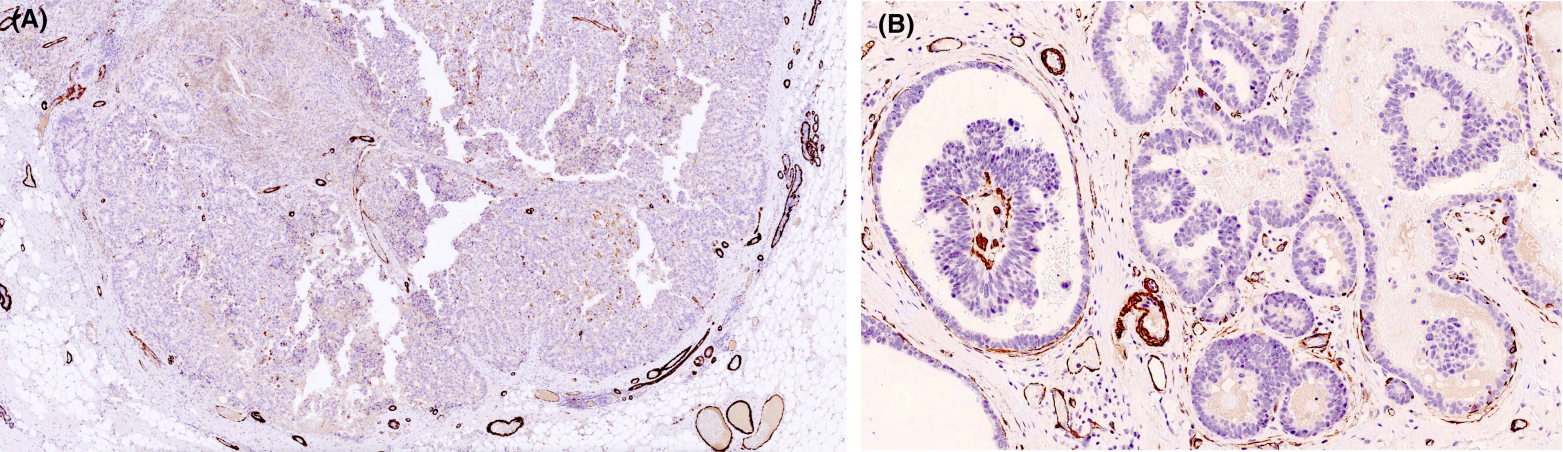
Myoepithelial cell (MEC) immunohistochemistry (IHC) (smooth muscle myosin heavy chain) showing the absence of MECs in the cores and at the periphery with a positive internal control around normal breast ducts and blood vessel walls (A). (B) A partial loss of MECs around DCIS papillary ductal profiles adjacent to the main EPC index lesion (smooth muscle actin). [Color figure can be viewed at wileyonlinelibrary.com]

EPC, defined as a papillary carcinoma with slender fibrovascular cores covered by neoplastic epithelial cells of low or intermediate nuclear grade, typically presents within a cystic space surrounded by a fibrous capsule of varying thickness^13^, ^14^, ^15^, ^16^, ^17^, ^18^, ^19^ (Figure 1). The neoplastic epithelial cells typically show diffuse, strong, oestrogen receptor (ER) nuclear positivity, and lack basal cytokeratin (CK) expression. The fibrovascular cores are usually devoid of an MEC layer. A peripheral MEC layer may be present but is frequently absent (Figure 2A). Some EPCs are associated with small, circumscribed, duct‐like, structures with a proliferation of uniform epithelial cells showing a cribriform or solid growth pattern, resembling Ductal carcinoma in situ (DCIS) but lacking peripheral MECs (Figure 2B). These foci are part of the EPC process rather than representing an associated invasive carcinoma. The latter displays an infiltrative architecture, usually with different morphology and associated with a desmoplastic stromal response. EPC with these morphological features, and in the absence of stromal invasion in the surrounding breast tissue, is staged as an *in situ* tumour (pTis),^13^ regardless of the presence or absence of a peripheral MEC layer.

#### Controversies and approach

##### The use of myoepithelial cell markers and receptor studies

Although the absence of peripheral MECs in EPC without frank stromal invasion is not used as a marker of invasion and these tumours are staged as *in situ* (pTis) disease, the routine use of MEC markers e.g. p63, smooth muscle actin (SMA, calponin, smooth muscle myosin heavy chain [SMMHC] is encouraged in the evaluation of possible EPC (Figure 2). The small proportion of tumours that maintain a peripheral MEC layer are considered biologically and clinically to represent *in situ* disease with no risk of metastasis. In EPC, although the expression of MEC IHC markers is absent in the majority of cases (40–90%), peripheral MECs may be detected using IHC as a continuous or an attenuated layer, or observed focally at the epithelial stroma interface in some EPCs.^15^, ^18^, ^20^ MECs may be positive for some MEC IHC markers but negative for others. Therefore, any IHC demonstration of MECs should be considered to indicate a peripheral MEC layer regardless of the extent or other negative MEC IHC markers. A pragmatic approach should be adopted in deciding the number of MEC markers and number of tumour blocks to be studied. There appears to be a reverse relationship between detection of MEC marker expression and the thickness and the degree of stromal/inflammatory reactivity of the peripheral capsule. EPC with a well‐defined margin lacking a thick reactive capsule tends to show MEC IHC marker expression more frequently than those with a thick reactive capsule (personal observation). This feature may be used to guide the number of MEC markers studied and the number of blocks evaluated. In general, it is recommended that at least two MEC IHC markers are studied on the most representative tumour block and, in occasional cases, two or three blocks can be stained.

EPC that lacks a peripheral MEC layer, as demonstrated using IHC, has low metastatic potential despite clinical staging as an *in situ* tumour.^14^, ^19^, ^20^, ^21^ It is advisable to include this information in the pathology report so that patients and their treating physician are aware of the low risk of metastases associated with these lesions that are staged as *in situ* disease to avoid overtreatment rather than to reflect their biology^20^ (EPC lacking MECs are recognized to have some metastatic potential, but the risk is too low to justify a diagnosis of invasive carcinoma and the current consensus opinion is to stage and manage these tumours as *in situ* disease; pTis). In addition, despite WHO guidance,^13^ the final classification of these lesions is based on the interpretation of a constellation of features that have some subjectivity and the concordance of classifying such lesions is not perfect.^11^

**Figure 3:**
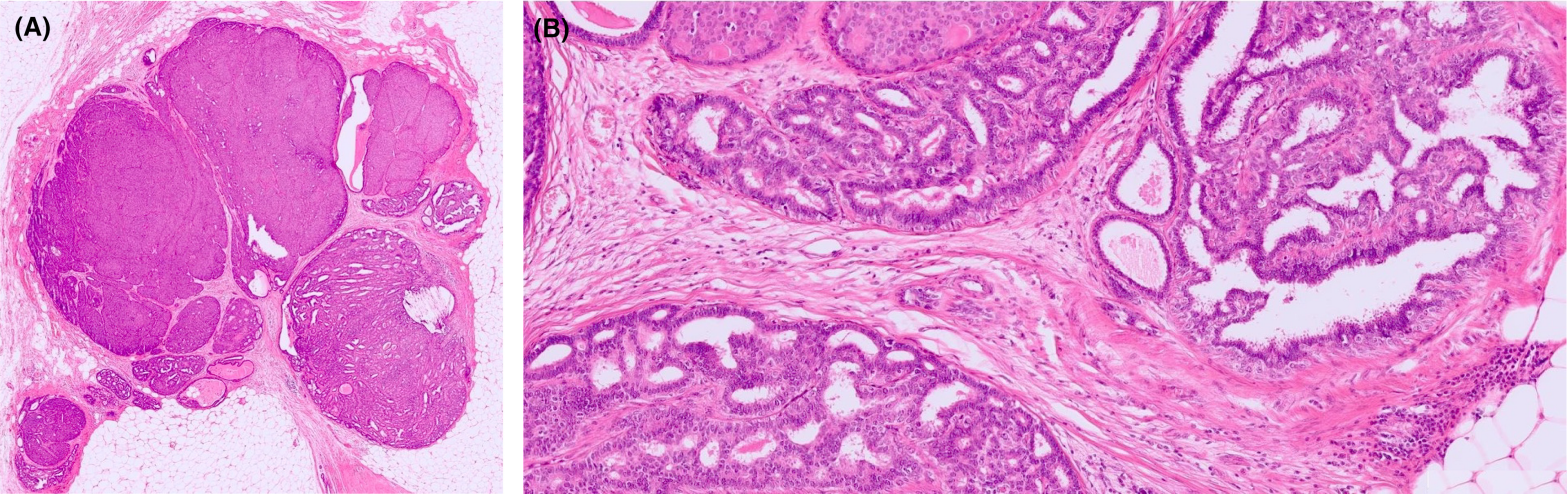
DCIS of solid and cribriform type colonizing a papillary lesion with subtotal involvement (A). Residual benign papillary lesion can be appreciated in the lower part of the figure and in (B). [Color figure can be viewed at wileyonlinelibrary.com]

MECs lining the fibrovascular cores are typically absent in papillary Ductal carcinoma in situ DCIS, EPC, and invasive papillary carcinoma. The demonstration of MECs lining the fibrovascular cores of a papillary carcinoma supports the diagnosis of an intraductal papilloma colonized by DCIS (Figure 3). The extent of colonization of papilloma by DCIS is inversely related to detectable MEC marker expression within the lesion. Some intraductal papillary tumours are composed of papilloma focally involved by neoplastic cells just sufficient for the classification of DCIS, while other tumours may comprise a predominant single population of neoplastic epithelial cells, with occasional residual MECS lining the fibrovascular cores and represent the other end of the spectrum of colonization. Regardless of the extent of residual papilloma and in the absence of any accompanying invasive disease, these tumours represent true *in situ* disease and have a superior prognosis to EPC with no risk of metastasis.

The combination of ER and a basal cytokeratin (e.g. CK14 or CK5/6) is useful to demonstrate the clonal nature of the epithelial cell component. ER is also recommended as a predictive biomarker in DCIS.^22^, ^23^ EPC has a higher risk of recurrence and nodal metastasis than conventional type DCIS^20^ and chemopreventive endocrine therapy may be indicated in some patients. EPC is also more frequent in elderly patients in whom endocrine therapy may offer alternative treatment if surgery is not feasible.^20^

**Figure 4:**
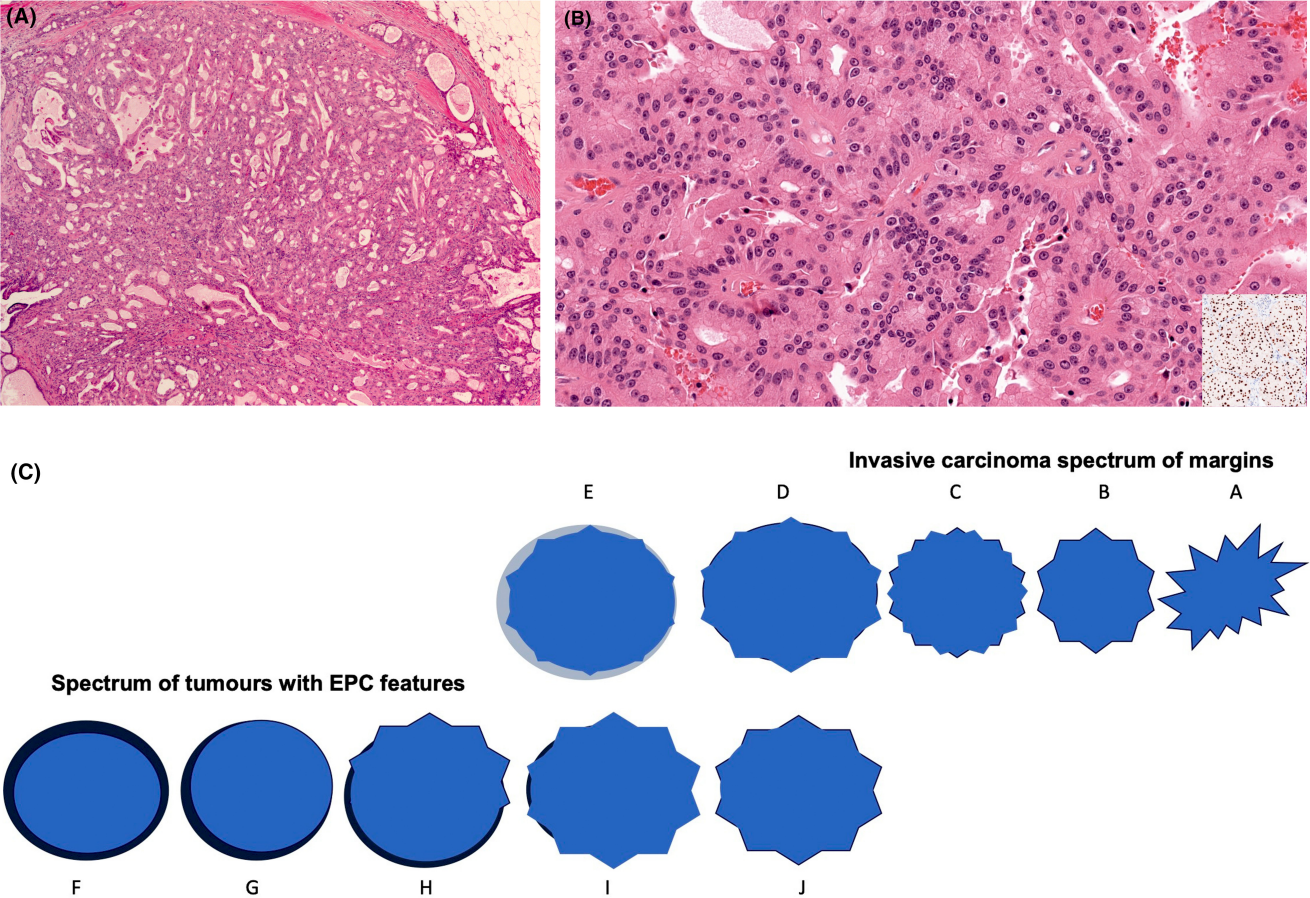
Apocrine EPC demonstrating lack of MECs throughout the lesion, a complex architecture with cribriform areas, and a thick fibrous capsule at the periphery (A). Neoplastic low‐grade apocrine cells may show ER positivity in addition to monomorphism of the cells contrasting with a hyperplastic apocrine proliferation (B; inset is ER staining). (C) The spectrum of invasive carcinoma margin from infiltrative stellate‐like tumours (A,B) to those circumscribed tumours with a pushing margin (D,E), which may be associated with a stromal response at the periphery mimicking EPC, at least focally (E). (F–J) The spectrum of tumours with EPC features from typical EPC with a circumscribed margin and a well‐developed peripheral capsule and low to intermediate nuclear grade features (F), with deficient capsule focally (G,H), to those that lose the well‐developed capsule and show infiltrative margins (I,J). These tumours (I,J) can be considered as invasive based on the pattern of marked stromal invasion or a combination of lack of MECs, high nuclear grade with or without an aggressive immunophenotype, and focal infiltration. Categorisation of such overlapping tumours (D,E,I,J) are challenging and further work‐up is advised. [Color figure can be viewed at wileyonlinelibrary.com]

An ER‐negative phenotype, similar to the presence of high nuclear grade features, in an EPC‐like tumour should prompt careful diagnostic consideration and further work‐up. ER negativity may be observed in papillary hidradenoma, which also lacks MECs but shows diffuse cellular expression of p63 and is typically superficial in location, and in intraductal papilloma with prominent apocrine metaplasia.^24^ The differential diagnosis also includes apocrine EPC,^25^ true ER‐negative EPC, which is relatively rare and EPC‐like invasive high‐grade IBC.^6^, ^26^, ^27^ Apocrine EPC that lacks definite stromal invasion is classified as EPC regardless of ER status or MEC layer^13^ (Figure 4). In ER‐negative EPC‐like tumours with high nuclear grade, high mitotic activity, and no peripheral MEC layer, it is helpful to perform GATA3 and SOX10 IHC to support primary breast origin as metastatic tumours with papillary morphology should be excluded. The use of Ki67 may also be used to highlight the proliferative activity of the tumour, which is usually increased in ER‐negative IBC.

There is currently no indication to assess HER2 status in EPC with classical histological features, as these are typically HER2‐negative^15^ and systemic therapy is not indicated. However, HER2 IHC may be used as a diagnostic marker to assist categorisation if EPC‐like IBC is suspected (an EPC‐like tumour lacks a peripheral MEC layer and shows high nuclear grade features, with or without ER negativity), in which case HER2 positivity (IHC score 3+), would further support a diagnosis of IBC.^18^, ^21^, ^28^, ^29^ There is no evidence to support staging HER2‐positive or triple‐negative high‐grade breast carcinomas that lack a peripheral MEC layer as *in situ* disease based on circumscription or papillary architecture, as these tumour phenotypes typically reflect biologically aggressive invasive disease (Figure 4C) and warrant adjuvant therapy. Although conventional type DCIS may show HER2 positivity, involved structures are surrounded by a peripheral MEC layer confirming its *in situ* nature.^30^, ^31^ EPC that maintains a peripheral MEC layer is an *in situ* carcinoma regardless of nuclear grade, ER or HER2 expression.

The use of biomarker status and histological nuclear grade, which represent a continuum in the multistep evolution of BC, in the evaluation of EPC and EPC‐like tumours may effectively result in anatomic restaging. However, in this context, these parameters are being utilized as diagnostic aids to assist categorisation and, in particular, to avoid underdiagnosis of EPC‐like IBC. EPC and EPC‐like IBC may show overlapping morphological features (Figure 4C) and the distinction may be challenging in view of the current approach to classification that considers EPC without a peripheral MEC layer as *in situ* disease. For example, a high‐grade circumscribed tumour that is triple‐negative or HER2‐positive is unlikely to display the indolent biological behaviour that is characteristic of true EPC.

**Figure 5:**
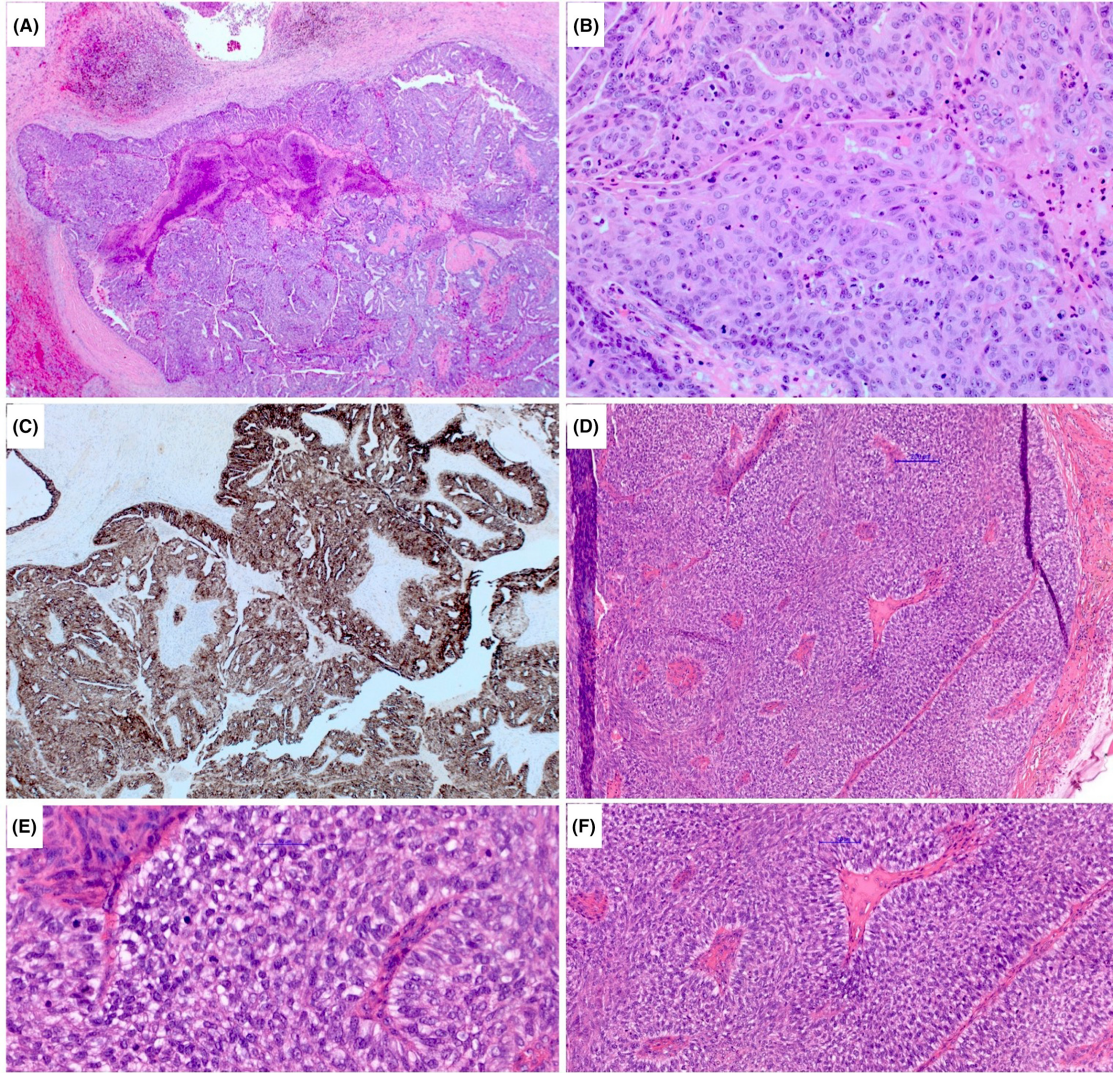
ER‐negative EPC‐like tumour (A and B) that shows strong diffuse basal CK expression (CK5/6 and CK14 in C) and bland cytological features with some squamoid differentiation (B). This tumour showed preservation of peripheral MECs. (D–F) Another ER‐negative EPC that featured bland cytological features and a basal phenotype (not shown) but lacked MECs. [Color figure can be viewed at wileyonlinelibrary.com]

We have encountered occasional EPC‐like tumours with a triple‐negative phenotype and strong expression of basal CKs, some with high mitotic counts. However, these rare variants of papillary carcinoma lacking high nuclear grade features and conventional type stromal invasion (Figure 5). The diffuse basal CK positivity, together with relatively bland cytological features, with some similarity to hyperplastic proliferative lesion cells, and the well‐defined tumour borders make a diagnosis of IBC less likely. It is our practice to report these low nuclear grade ER‐negative papillary lesions as a basal‐like variant of EPC and stage them as *in situ* carcinomas similar to the more typical forms of EPC.

##### Identification of microinvasion and associated invasive carcinoma in EPC

EPC may be associated with two types of invasive carcinoma; a separate component of coexistent IBC or a focus of invasion arising from the EPC.

**Figure 6:**
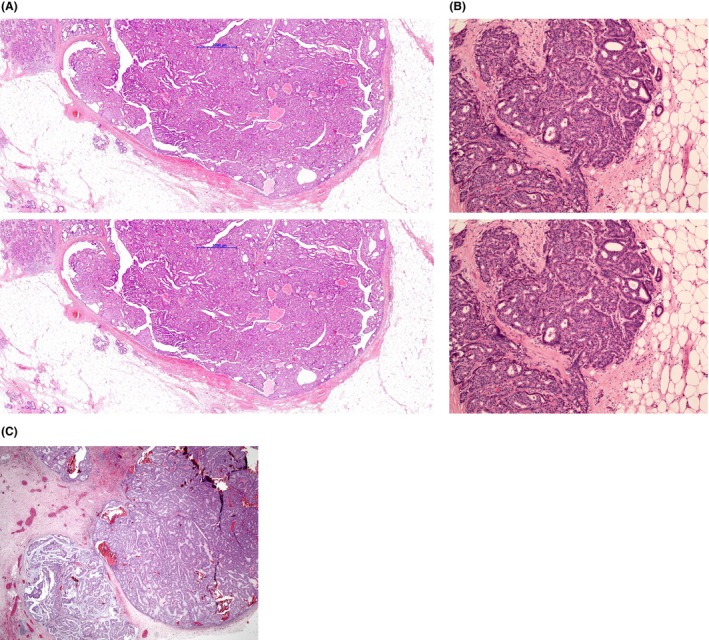
(A) EPC with a focus of invasive NST carcinoma in the upper left part of the picture. (B) A small focus of invasive carcinoma with preservation of the papillary morphology. (C) A distinct small focus of invasive carcinoma with mucinous features adjacent to EPC. [Color figure can be viewed at wileyonlinelibrary.com]

In the first scenario, the invasive carcinoma may show different morphology to the EPC, e.g. invasive lobular carcinoma, invasive micropapillary carcinoma or, more frequently, no special type (NST) (Figure 6A,B). Mucinous carcinoma may be seen in association with EPC (Figure 6C) but is more common in solid papillary carcinoma (SPC). Assessment of prognostic and predictive variables is performed on the invasive component with the EPC considered to represent an *in situ* component of the tumour. If the invasive focus is located >5 mm away from the EPC, it can be considered as a separate focus of invasion not related to or arising from the index EPC, as per conventional type carcinoma focality assessment.^13^

**Figure 7:**
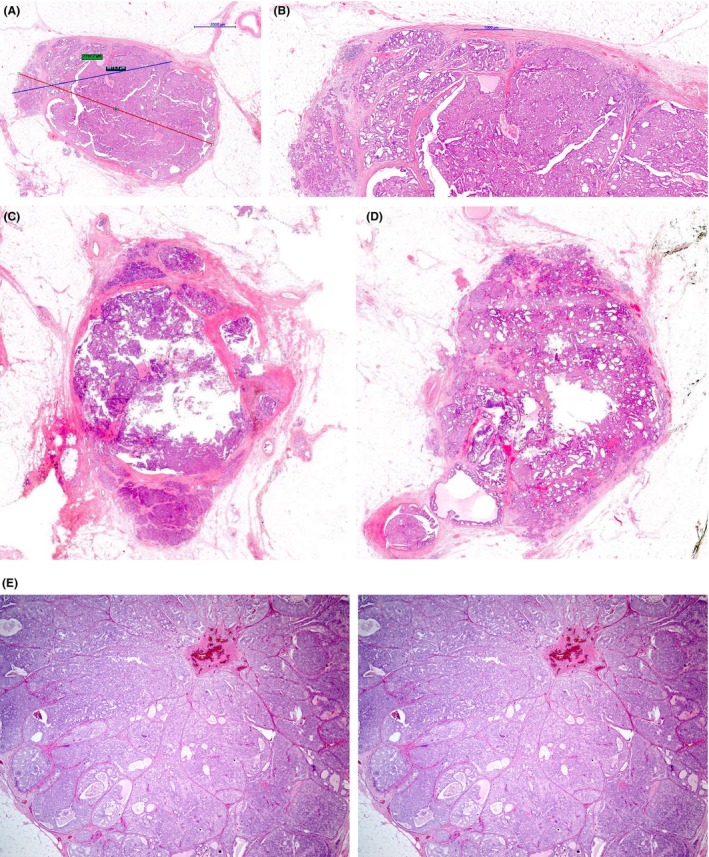
EPC that is partly invasive (7.7 mm in size marked by the blue line) and partly *in situ*; whole tumour size is 9.8 mm (red line) (A). (B) Details of the area of invasion with definite stromal invasion, with mixed NST and invasive papillary morphology. (C,D) Two invasive papillary carcinomas with some EPC features in which the whole tumour is considered as invasive for management purposes. (E) Part of an invasive papillary carcinoma (confluent mass of DCIS‐like expanded ducts mimicking EPC/SPC) with a jigsaw pattern, similar to a solid papillary carcinoma invasive pattern, and lack of expression of MEC markers, which is an essential criterion to consider such a tumour as invasive. [Color figure can be viewed at wileyonlinelibrary.com]

**Figure 8:**
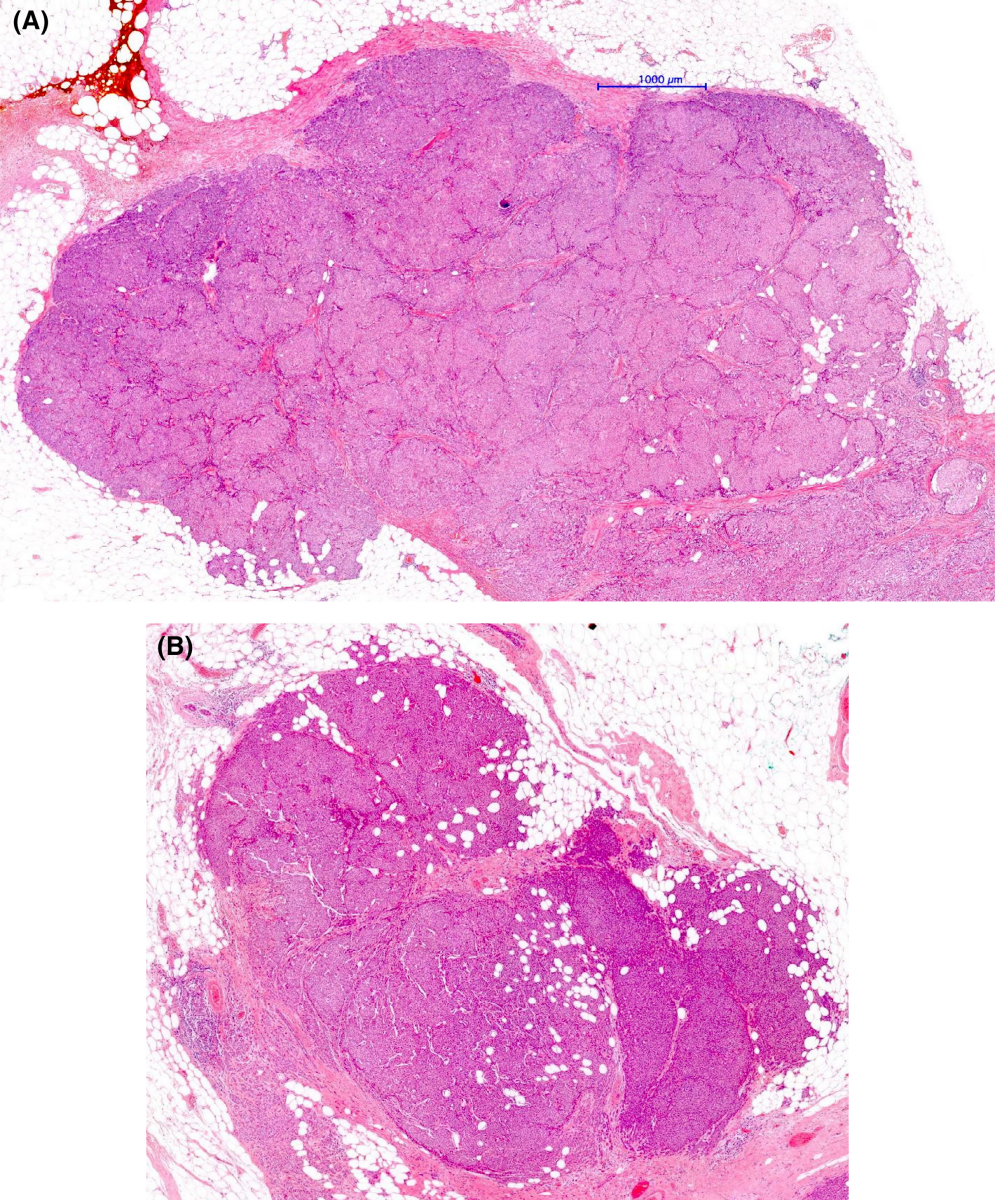
A and B: Invasive papillary carcinoma with an EPC‐like pattern, but definite infiltration of fat is evident with the tumour. [Color figure can be viewed at wileyonlinelibrary.com]

In the second scenario, i.e. where invasive carcinoma arises from the EPC, it may be difficult to assess the extent of invasion and to decide whether the entire EPC is invasive (Figure 7). Frank invasion is defined as the presence of neoplastic elements that permeate beyond the fibrous tissue capsule with an irregular infiltrative appearance.^13^ In the presence of frank invasion, the Nottingham grade, tumour size (TNM T stage), and receptor status should be assessed in the frankly invasive component. The size of EPC should be considered in the assessment of the whole tumour size. The presence of entrapped fat cells within an EPC is a definite sign of invasion (Figure 8) and the entire lesion is considered to be invasive for the assessment of prognostic and predictive variables. If lymphovascular invasion (LVI) is observed in association with a tumour that displays EPC features, additional work up, including extra‐level sections and further tissue sampling to identify an invasive focus, is required.

**Figure 9:**
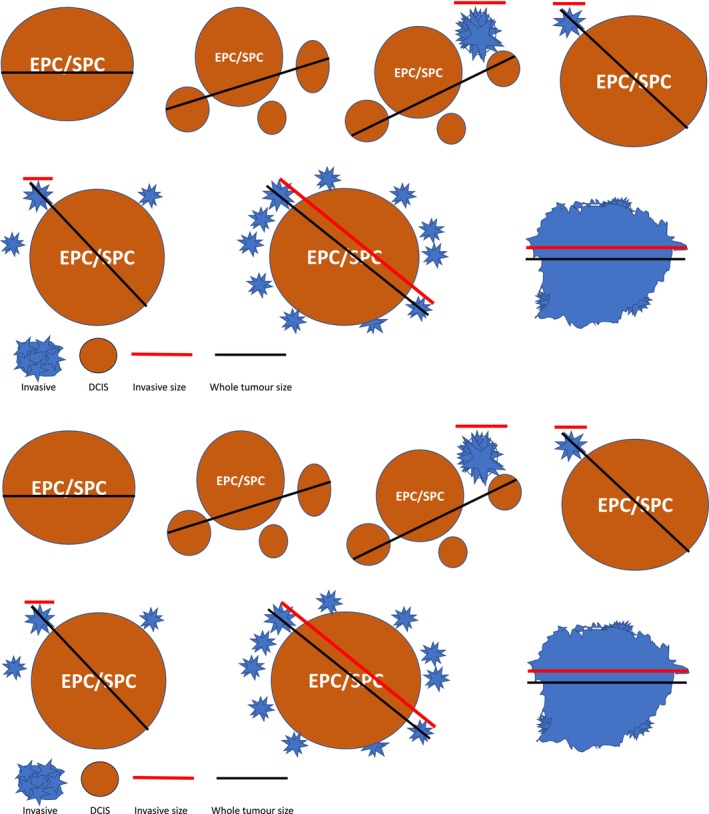
Guide for assessment of size in EPC (and SPC) with or without invasion. Satellite foci of invasion are measured separately. Pathological stage (pTNM and/or NPI) is based on the size of the largest invasive focus. Pure DCIS and papillary carcinoma *in situ* are graded accordingly to the nuclear characteristics, and pathological stage is pTis. In DCIS or papillary carcinoma *in situ* associated with invasion, the invasive component is graded using the Nottingham criteria and pathological stage depends on the size of the invasive component. If the whole EPC is invasive, it is evaluated like conventional invasive breast carcinoma. [Color figure can be viewed at wileyonlinelibrary.com]

Accurate assessment of invasive tumour size may be challenging in EPCs with more than one focus of accompanying invasive carcinoma, particularly if these are separately located at the periphery of the tumour rather than forming a discrete invasive component (Figure 7A, B). A guide to measurement is illustrated in Figure 9.

**Figure 10:**
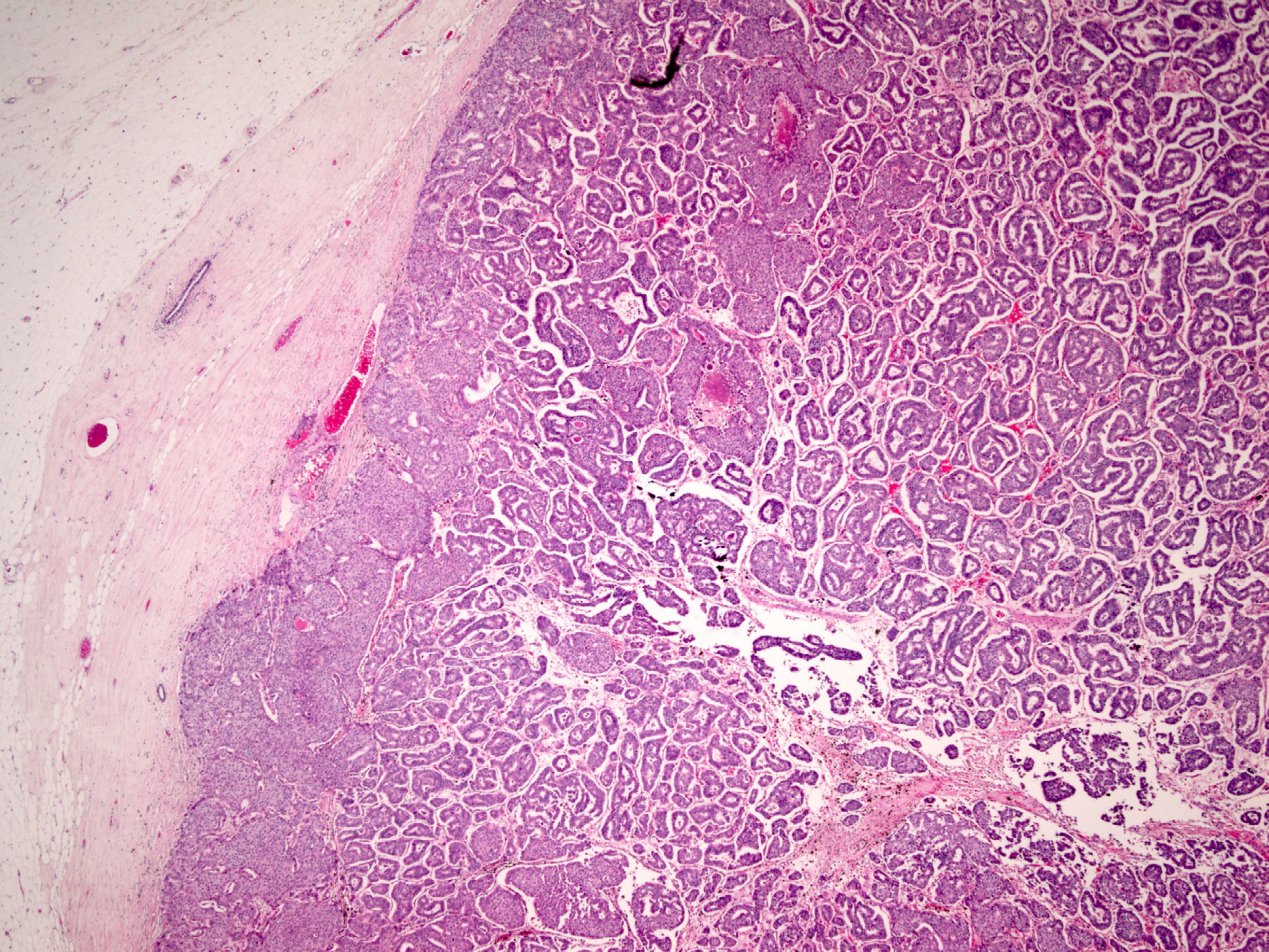
EPC‐like invasive tumour with an invasive micropapillary carcinoma component. Although the margin is well circumscribed, a thick peripheral fibrous capsule is lacking. [Color figure can be viewed at wileyonlinelibrary.com]

**Figure 11:**
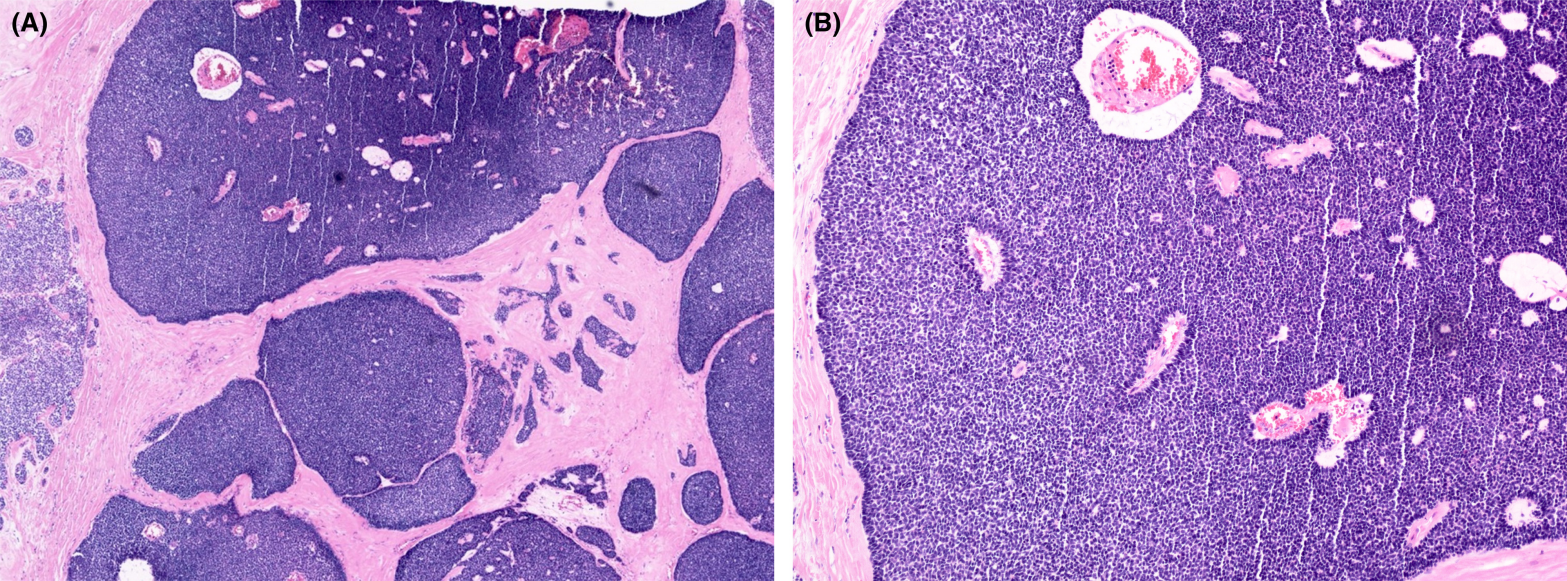
Solid adenoid cystic carcinoma (salivary gland‐like tumour) with large circumscribed nodules (A) showing a papillary architecture (B) mimicking EPC. The cytological (basaloid) features of these tumours and the characteristic IHC (basal‐like) profile (not shown in the picture) are the clues to the diagnosis. [Color figure can be viewed at wileyonlinelibrary.com]

EPC should also be differentiated from EPC‐like invasive lobular carcinoma with papillary morphology.^32^ These tumours are E‐cadherin‐negative and lack MECs. Florid, mass forming, Lobular Carcinoma in situ may overlap with EPC and invasive papillary lobular carcinoma but maintains MECs at the epithelial stroma interface. EPC lacking MECs and showing an invasive micropapillary carcinoma component should be considered as invasive carcinoma even if no conventional stromal invasion is seen^7^ (Figure 10). EPC should also be distinguished from the solid variant of adenoid cystic carcinoma with a papillary‐like architecture and pushing margin (Figure 11). These tumours show basal cytokeratin and/or p63 expression and are frequently CD117‐positive and ER‐negative.

**Figure 12:**
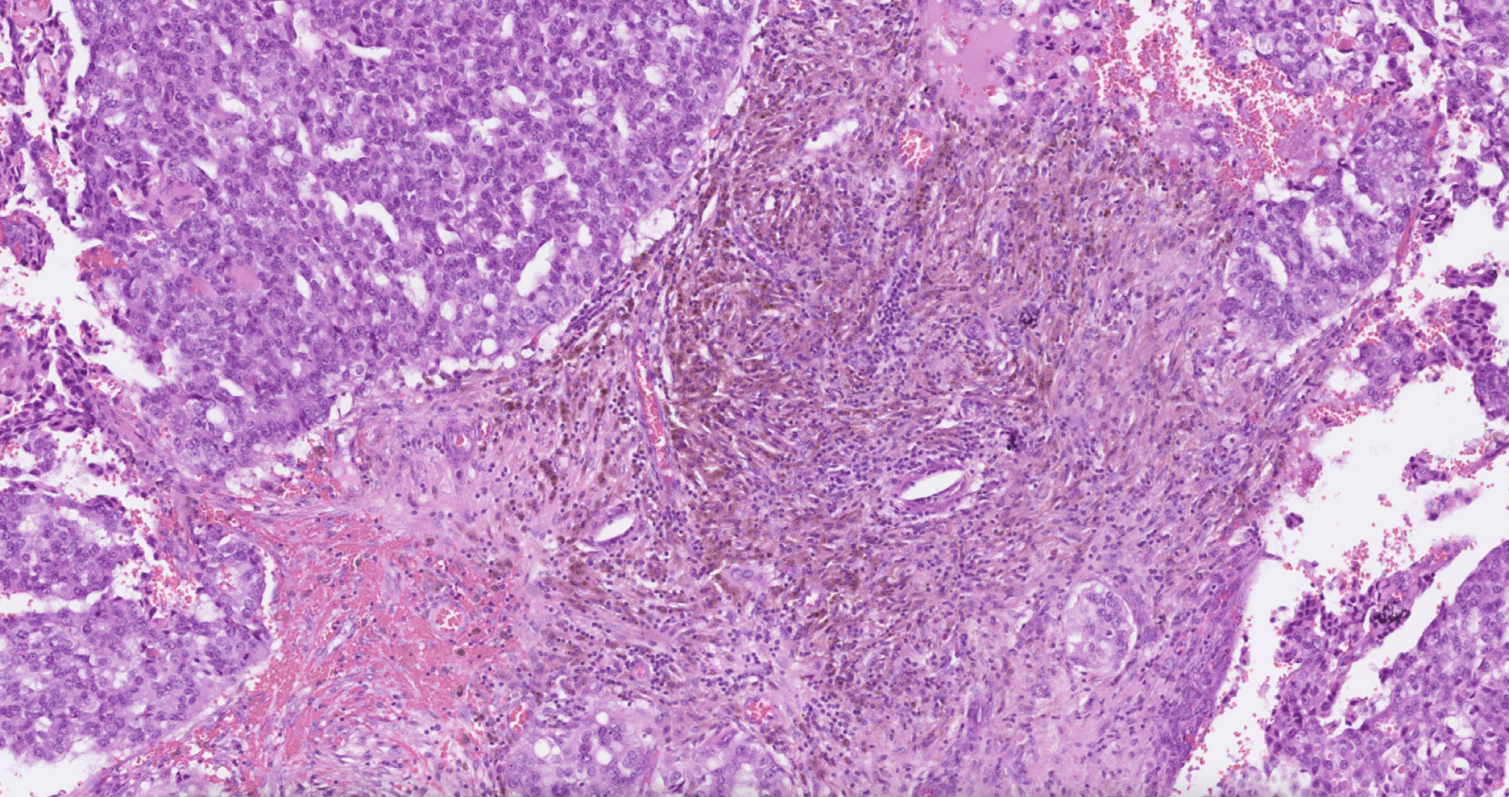
Core biopsy‐related changes with a florid fibrous tissue reaction showing evidence of recent haemorrhage (hemosiderin deposition). These changes are typically localized and may show disruption of the tumour architecture at the side of the biopsy‐related trauma. [Color figure can be viewed at wileyonlinelibrary.com]

True invasion must be differentiated from entrapment of neoplastic cells within the fibrous capsule and from epithelial cell displacement within the biopsy site scar tissue. Many EPCs are surrounded by zones of reactive fibrosis,^14^ frequently with foci of chronic inflammation and recent or resolving haemorrhage likely secondary to repeated minor rupture due to increased intraluminal pressure following distal duct obstruction (Figure 12). Small papillary or tubular structures may be observed within these areas of inflammation and haemorrhage, usually within the fibrous capsule, and represent entrapped epithelium and not true invasion. In some tumours, particularly following recent core needle biopsy (CNB), entrapped epithelium may be observed outside the fibrous capsule leading to concern regarding invasion. Appreciation of the accompanying tissue changes, including early fibrosis and haemorrhage, helps to avoid overdiagnosis of invasion and distinction from infiltrative papillary carcinomas, that either show a jigsaw growth pattern or diffusely infiltrative papillary cystic structures, that likely represent invasive carcinoma with preservation of the cystic papillary configuration. If in doubt regarding entrapped cells within or at the periphery of the capsule, it is advisable to categorize as EPC with microinvasion, or with a focus uncertain for invasion if the epithelial entrapment exceeds 1 mm size, to reflect an increased risk of disease progression.

The presence of multiple foci of invasion at the periphery of an EPC suggests that the whole tumour represents invasive carcinoma with an EPC‐like pattern. Epithelial cell displacement may also be seen in blood vessels mimicking lymphovascular invasion, and even in regional lymph nodes.^33^, ^34^, ^35^, ^36^ Such findings should prompt further evaluation of the lesion to exclude the possibility of invasion and prior to staging as *in situ* disease. In the absence of true invasion, the finding of epithelial cell displacement, even within lymphatics or regional lymph nodes, does not alter classification of the index lesion. Consideration of the time interval between biopsy and excision may also help to clarify the significance of the findings and, in the case of displacement, is usually <4 weeks.^33^, ^34^, ^35^, ^36^ Displaced neoplastic cells degenerate over time, while true invasive cells continue to proliferate and may display different morphology to the EPC, as outlined earlier. Consultation with colleagues and/or external referral is encouraged in these rare cases.

Although not EPC, pathologists should be aware of the difficulty in distinguishing papillary DCIS from some forms of invasive papillary carcinoma and tall cell carcinoma with reversed polarity (TCCRP). Papillary DCIS is a morphological subtype of DCIS featuring fibrovascular cores lined by neoplastic ductal epithelium and devoid of MECs, but with a preserved peripheral MEC layer and well‐defined duct profiles.^13^ Some invasive papillary carcinomas may present as papillary clusters of variable size mimicking papillary DCIS, but these typically lack a peripheral MEC layer and show disruption of the duct profile, at least focally. Further examination typically reveals definite stromal invasion around some of these DCIS‐like duct profiles. TCCRP lacks MECs and shows reversed polarity of the cells facing the stroma, together with other defining features including ER negativity and expression of basal cytokeratins.

##### High‐grade tumours with EPC‐like pattern

**Figure 13:**
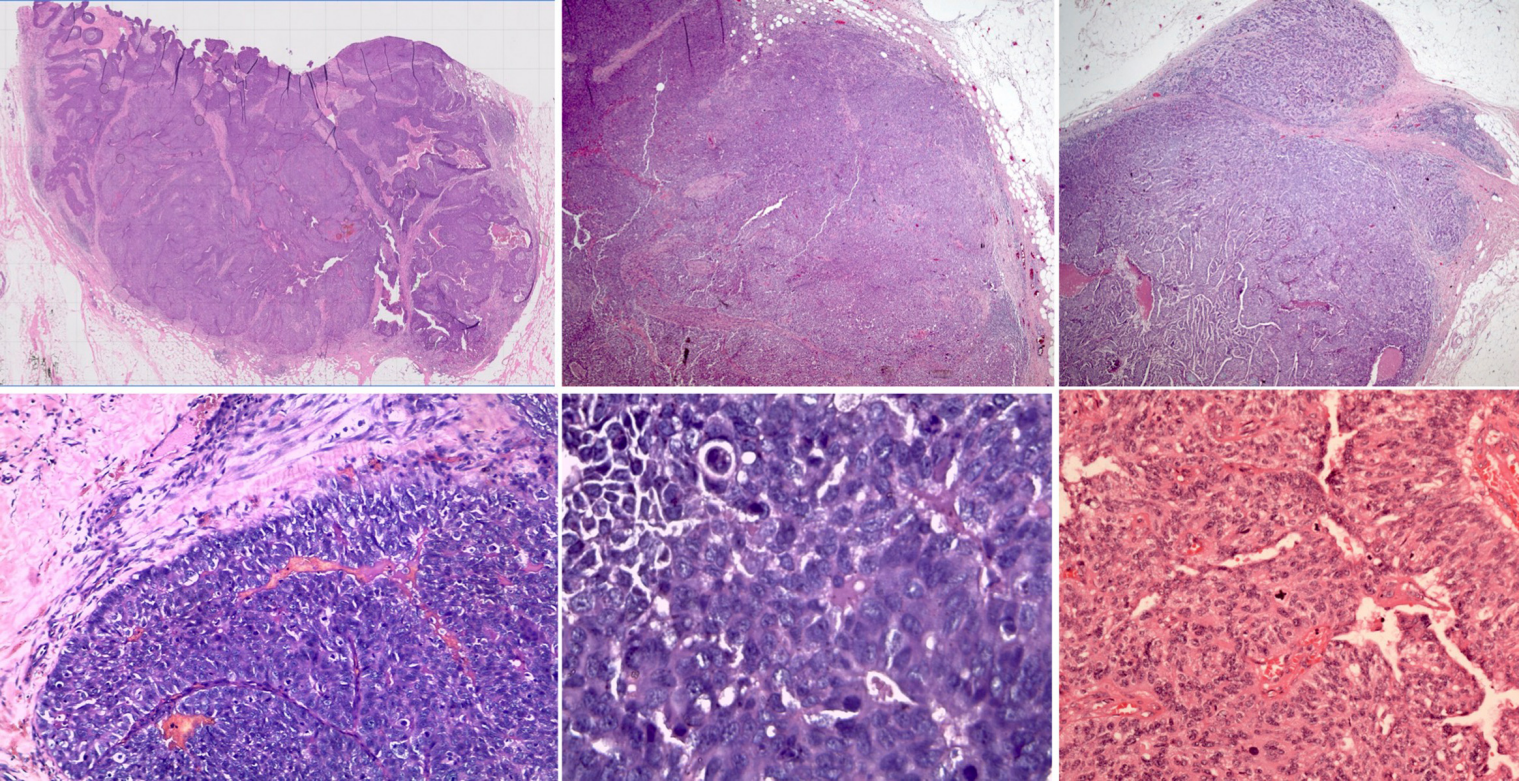
Various examples of high‐grade invasive breast carcinoma with an EPC‐like growth pattern. High nuclear grade features, mitotic activity, definite stromal invasion focally, and the absence of the characteristic thick fibrous capsule at the periphery of most of the lesion. [Color figure can be viewed at wileyonlinelibrary.com]

**Figure 14:**
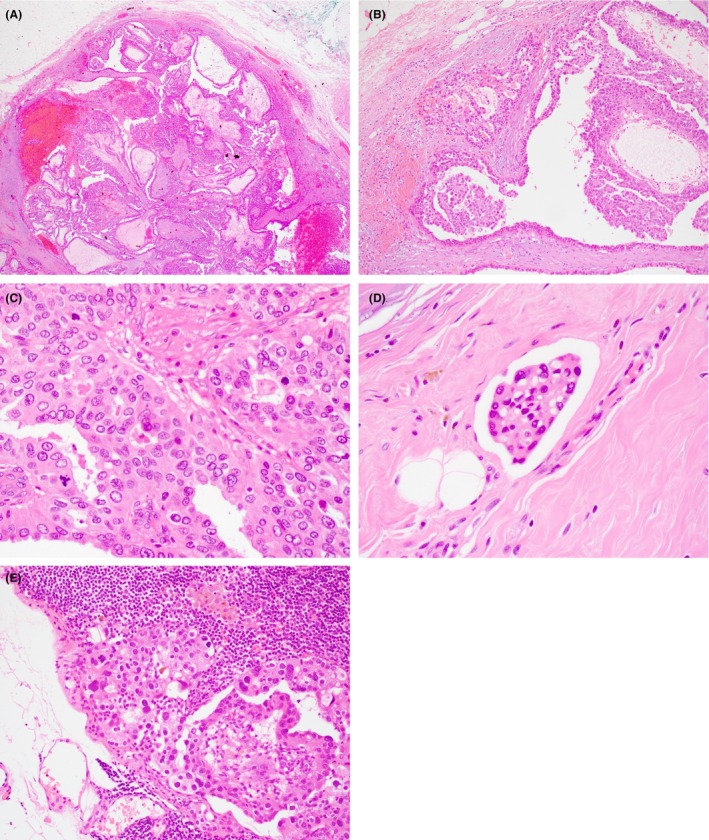
An EPC‐like IBC showing a well‐developed papillary configuration (A), infiltrative outline and high‐grade cytology (B), increased mitotic activity (C), lymphovascular invasion (D), and lymph node metastasis (E). [Color figure can be viewed at wileyonlinelibrary.com]

Papillary DCIS and EPC with a preserved peripheral MEC layer constitute *in situ* carcinoma regardless of nuclear grade or receptor status. Invasive papillary carcinoma, like papillary DCIS, may also be high grade. As referred to earlier, it is advisable to exert caution before staging a circumscribed, high nuclear grade tumour that lacks a peripheral MEC layer at the epithelial stroma interface, as *in situ* carcinoma, as this may result in undertreatment of high‐grade IBC.^11^ High grade, highly proliferative, invasive tumours, particularly those with triple‐negative or HER2‐positive biology, may have pushing/circumscribed margins^6^, ^26^, ^27^ with entrapped stromal tissue mimicking a papillary architecture^37^ (Figures 13 and 14). In a previous study,^6^ we observed tumours with a papillary architecture, cystic configuration, and no peripheral MECs, of large size with high nuclear grade and high mitotic figures, variously triple‐negative and ER‐positive, (mis)classified as *in situ* carcinoma (EPC). We have also reported an example of high‐grade carcinoma with a circumscribed margin and papillary architecture on CNB, designated EPC. On excision, the tumour showed focal but definite stromal invasion, lacked a well‐developed fibrous capsule despite the circumscribed margin, and displayed a triple‐negative phenotype. The papillary cores were mainly poorly formed and overlapped with entrapped stromal tissue. The tumour also showed focal areas of geographic necrosis mimicking basal‐like IBC. In some independent studies, high‐grade, EPC‐like, tumours exhibited prominent lymphoplasmacytic infiltrates, more crowded and thicker papillae, and larger tumour size (>4 cm).^16^, ^17^ Definite infiltrative components were identified in 80% of the reported cases.^17^ High‐grade EPC‐like tumours tended to be negative for hormone receptors, positive for basal‐like markers, including CK5/6, and showed a significantly high Ki‐67 index.^16^, ^17^ These tumours should be graded, staged, and managed as IBC.^6^

Pathologists should also avoid overclassifying typical EPC with intermediate nuclear grade features as invasive carcinoma. Definition of high nuclear grade can vary, and defining criteria may be applied differently by pathologists resulting in upgrading some typical EPCs with intermediate nuclear grade or with occasional scattered pleomorphic cells. The cytology of invasive, high‐grade, EPC‐like tumours should be similar to that of high nuclear grade DCIS and other high‐grade NST and basal‐like IBCs.^38^ In addition to an increased nucleo‐cytoplasmic ratio, marked nuclear enlargement (>2.5 times the size of normal epithelial cells), irregular nuclear membrane, variation in size and shape (pleomorphism), and hyperchromatic vesicular nuclei with prominent nucleoli should also be present.^17^, ^38^ These tumours typically show high mitotic counts with mitotic activity score 2 at least.^38^ Classification of rare circumscribed high‐grade tumours showing ER‐positive and HER2‐negative phenotype together with other features of EPC, including well‐developed fibrous capsule and papillary architecture throughout the lesion, is challenging. Further workup and specialist opinion should be pursued in such cases before final classification.

##### Core needle biopsy and excision specimens

###### Core needle biopsy

A CNB specimen that includes elements of a typical EPC with a well‐defined margin and a thick peripheral capsule, with or without a peripheral MEC layer, is considered an *in situ* tumour and classified as B5a with recommendation for wide local excision if there is good radiology‐pathology concordance.^39^ Surgical practice varies regarding sentinel lymph node (SLN) biopsy in the management of these tumours (see below). The diagnostic work‐up should include ER, basal CKs, and MEC IHC. Assessment of HER2 status in typical EPC tumours is not required, either as a predictive marker or as a prognostic marker.

High nuclear grade tumours with an EPC growth pattern and positive MEC markers at the periphery of the lesion are reported as high‐nuclear grade EPC (B5a), irrespective of the receptor status. The possibility of false‐negative staining of MEC markers, in addition to the adequacy of sampling, should be considered in the work‐up of this type of tumour.

A malignant tumour, with some EPC‐like morphology, which lacks peripheral MECs and shows stromal infiltration, or is accompanied by a definite invasive carcinoma component (e.g. NST, lobular or mucinous), is reported as IBC (B5b^39^). Receptors, including ER and HER2, should be assessed and excision with SLN biopsy advised. Discussion of neoadjuvant therapy may be considered, depending on the characteristics of the invasive component.

If the “EPC” shows high nuclear grade and frequent mitoses, in addition to the lack of the peripheral MEC layer, the tumour likely represents high‐grade IBC with an EPC‐like pattern. Further diagnostic work up includes additional MEC IHC and ER, PR and HER2 studies. Ki67 may highlight increased proliferative activity. It is our observation that EPC typically shows low Ki67 index while high Ki67 expression is more common in high‐grade EPC‐like, likely, invasive tumours. Additional levels may be useful in demonstrating more conventional stromal invasion. In high‐grade papillary carcinoma that lacks MEC marker expression, particularly ER‐negative or weak, exclusion of metastatic papillary carcinoma such as papillary serous carcinoma should also be considered.

**Figure 15:**
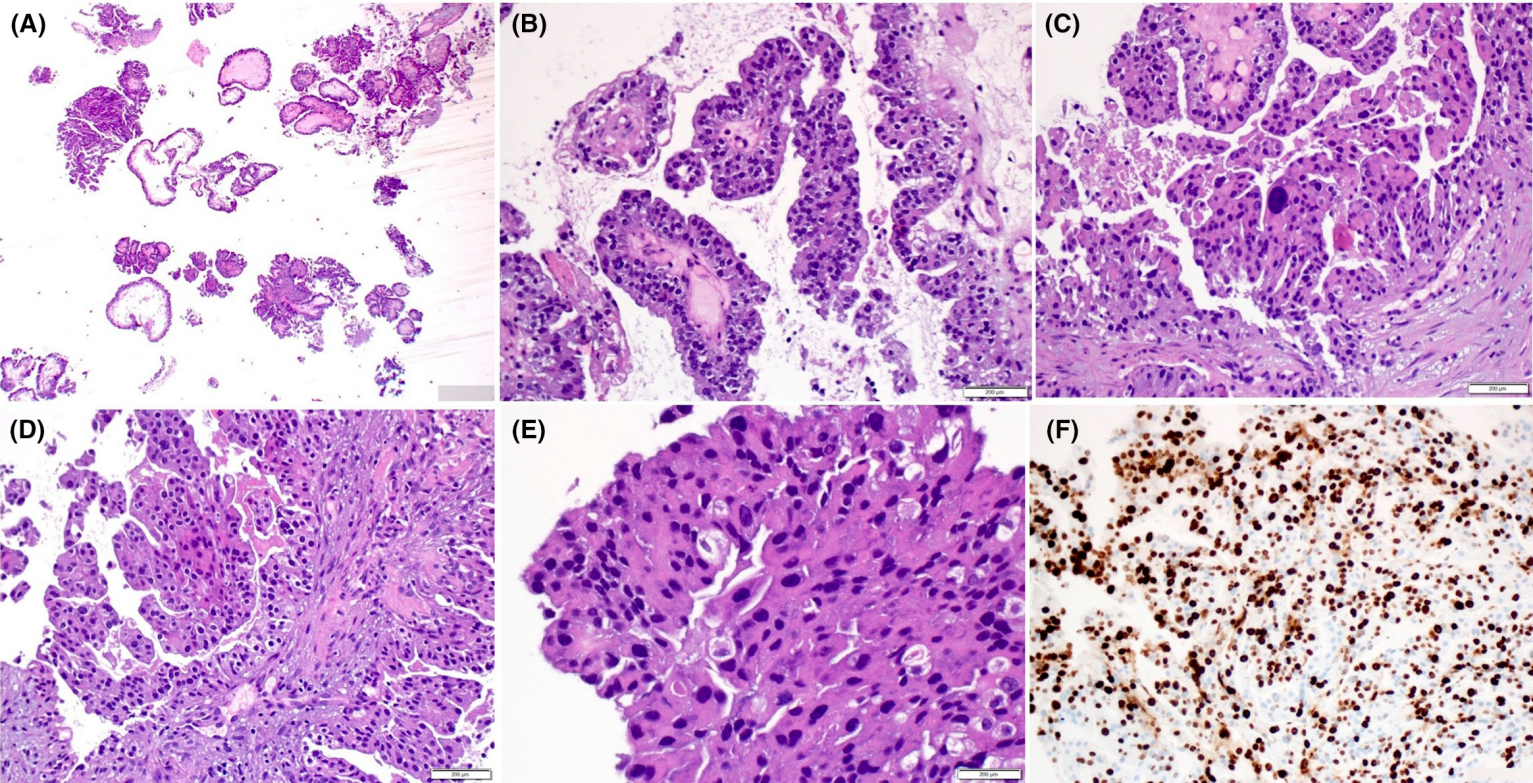
Core needle biopsy showing parts of a malignant papillary lesion with papillary cores lined by single and multiple layers of neoplastic cells (A–D) displaying high nuclear grade (E) and high Ki67 index (F). A large panel of myoepithelial cell markers were negative, but the tumour lacked the peripheral stroma interface to comment on the existence of a peripheral myoepithelial cell layer. The neoplastic cells showed triple‐negative phenotype, but additional markers confirmed breast origin (GATA3 and SOX10‐positive but negative for PAX8, WT1, and TTF1). This case is categorized as B5c. [Color figure can be viewed at wileyonlinelibrary.com]

In our opinion, EPC lacking peripheral MECs and showing an irregular margin or high nuclear grade features can be reported as B5c,^39^ reflecting uncertainty regarding the possibility of invasion with a recommendation to fully excise with SLN biopsy but not for consideration of neoadjuvant therapy. Similarly, CNB with fragments of high‐grade, ER‐negative and/or HER2‐positive EPC‐like tumours that lack peripheral epithelial stroma interface and, therefore, not possible to evaluate the existence of the peripheral MEC layer or stroma invasion in the cores (Figure 15), are best categorized as B5c. Ideally, surgical excision and SLN biopsy should be performed initially, permitting further evaluation of the tumour prior to decisions regarding adjuvant treatment. Neoadjuvant therapy may be considered for patients with triple‐negative or HER2‐positive high‐grade EPC‐like tumours that lack a peripheral MEC layer and show evidence of stromal invasion (B5b). However, the level of confidence in making a definite diagnosis on the CNB should be clearly presented at MDT.

A potential pitfall is metastatic serous carcinoma of ovarian or peritoneal origin that may display morphological overlap with EPC on CNB. These tumours show high nuclear grade features with occasional bizarre nuclei, are strongly positive for PAX8, p53 and WT1, and negative for GATA3 and SOX10 despite showing a CK7+/CK20− phenotype similar to breast tumours. They may show ER positivity.

###### Excision specimen

The tumour should be processed in its entirety for microscopic evaluation and MEC markers performed. Infiltrative foci will usually be seen in invasive EPC‐like carcinomas even if large areas of the tumours show a circumscribed margin. An abrupt transition of morphology between EPC and invasive areas is likely to be seen in mixed *in situ* and invasive carcinomas (e.g. focal progression of *in situ* carcinoma to invasive disease), while a gradual transition between circumscribed and infiltrative foci (intermingled areas without sharp distinction in the change of the pattern) is more common when the entire lesion is invasive. Most well‐circumscribed EPCs lack a peripheral MEC layer. Focal retention of MECs at the epithelial stroma interface in tumours with mixed circumscribed and infiltrative areas provides evidence of a definite *in situ* component with the infiltrative foci classified as IBC due to their configuration. Focal retention of MECs at the epithelial stroma interface in tumours with circumscribed margins provides evidence of a definite *in situ* nature except if focal areas of stromal invasion is identified. When the whole tumour is invasive, MECs are typically absent throughout. A final categorisation is rendered following consideration of morphology with particular emphasis on tumour outline and nuclear grade together with the results of MEC marker and biomarker studies.

##### Sentinel lymph node biopsy

The practice of sentinel lymph node (SLN) biopsy in EPC diagnosed on CNB varies. It is our approach to recommend SLN in EPC diagnosed on CNB even if no definite stromal invasion is seen similar to the approach for mass forming DCIS. In previous studies of EPC, invasion was detected in 41% (19/46),^21^ 44% (11/25),^16^ and 40% (12/30)^21^ on the excision specimen. Lymph node metastases were detected in 7.7% (3/39) of low and intermediate grade ER‐positive EPCs.^18^ In a follow‐up study of 29 patients (mean 47 months), five developed local recurrence (17%) and two distant metastases (7%), supporting the role of SLN biopsy in the management of EPC. In another study of 37 patients who underwent axillary staging, only one patient (2.7%) with EPC had evidence of nodal metastasis.^40^

###### Comment

Challenging cases should be reported with a brief explanation of the specific diagnostic challenge with a comment noting that concordance of classification of these tumours is not perfect, advocating a pragmatic approach to patient management in situations where there is doubt regarding the precise diagnosis. Specialist opinion should be sought in challenging cases. Radiation therapy as for DCIS and IBC following breast conservation surgery and clinical/radiological follow‐up is indicated.^22^, ^23^ Hormone therapy for ER‐positive tumours can be justified.^23^ Recommendations regarding adjuvant chemotherapy depends on the nature and biological profile of any accompanying invasive component and should be subject to MDT review. A similar approach can be adopted for the management of rare tumours that lack evidence‐based data regarding the clinical behaviour or treatment response, e.g. malignant adenomyoepithelioma *in situ*.^41^ We should be mindful that high nuclear grade EPC‐like tumours are likely to represent invasive carcinoma with potential for biological progression. ECP specific clinical trials to develop a reliable evidence base and precision medicine tools are warranted.

## Author contributions

EAR: prepared the manuscript, amended and updated it and approved the final version. CQ: Reviewed the manuscript and approved the final version

## Conflicts of interest

None.

## Data Availability

Data sharing not applicable to this article as no datasets were generated or analysed during the current study.
